# Foreign

**Published:** 1840-06-27

**Authors:** 


### FOREIGN.

### ANDRAL'S LECTURES ON THE ALTERATIONS OF THE BLOOD. NO. II.

*Disorders produced by Anemia in the functions of Nutrition.*

*Digestion.*—The process of chymification in a healthy man is arrested by a blood-letting. Digestion is deranged in those affected with anemia; they are subject to nausea, vomiting, dyspepsia, &c. Exhalations of gas are frequently met with in this affection, as well as in hysteria.

*Circulation.*—Syncope; palpitations of the heart, with acceleration of the strokes of the pulse; action of the heart irregular, tumultuous. Bruit-de-diable in the carotids is one of the best distinguishing characters of anemia, or chlorosis. This sound is not found in the heart. If the bellows sound exists in the heart, at the same time that it is found in the carotids, it does not furnish us with a pathognomonic symptom of chlorosis, as, in this instance, it depends upon disease of the heart.

The capillary vessels are subject to passive congestions. In animals destroyed by anemia, we find the conjunctiva of a bright red colour. Amaurosis following anemia, is accompanied with injection of the conjunctiva, hæmorrhages of the capillary vessels, epistaxis. These hæmorrhages cannot be combated by antiphlogistic remedies.

*Respiration.*—Panting, dyspnœa. Auscultation sometimes gives us no results; at other times we discover pulmonary congestions, or œdema of the lungs, which are attended with a crepitus. Sanguineous congestions are produced in animals by anemia, or the loss of blood.

*Secretions.*—Urine pale, transparent, very watery.

*Saliva.*—The mouth often presents a dryness which might lead us to attribute it to an irritation of the stomach; but such is not the case.

*Perspiration.*—By bleeding a man, we make him perspire: in persons suffering from anemia, sweating is brought on by very slight causes. The exhalations from the serous surfaces are frequently increased, and dropsies are the consequence. Anasarca is often produced in children after losses of blood, or chronic diseases. During a famine which prevailed in France, dropsy appeared in an epidemic form. Whenever the blood becomes impoverished, serous effusions show themselves; the blood being deteriorated, and the solids not receiving blood capable of repairing their losses, we should expect to find all the tissues of the body atrophied; but we do not find this to take place, except in the muscular system, whose nutrition is impaired, while that of the brain, liver, skin, &c., do not seem to suffer. The transparent cornea gives way in animals who have suffered great loss of blood. Anemia is favourable to the development of tubercles.

Anemia is chronic or acute. When spontaneous it is generally chronic; beginning with derangement of some of the functions either of digestion or the nervous system, with intermissions; the patient being one day better, another worse. Acute anemia resembles a disease in progress. [Case: A. B. had headach; palpitations of heart; debility; chlorotic tint; vertigo; pulse 120; frequent faintings; appearance of fever towards night; with symptoms resembling a cerebral affection, for which this case was mistaken, and treated by bloodletting, during which syncope came on. When he was put upon large doses of iron, he was speedily cured.]

Anemia may end in health or death. Simple anemia may be fatal from the impoverishment of the blood, and death may take place during the debilitated state, attended by symptoms of nervous disorder, or pulmonary congestion.

2. *Alterations of the Blood as to quality.*

Alterations in its chemical or physical constitution may exist, which are inappreciable to all our means of research.

Some of the alterations of its physical qualities may be detected by the sight, &c., without the aid of instruments; others are only discoverable through the aid of the microscope.

*First Section.—Physical alterations appreciable to our Senses.*

*Colour.*—Its dark colour may become lighter, on account of its deficiency of colouring matter, as is seen in lymphatic and chlorotic persons. Venous blood may assume a redder tint, and be mistaken for arterial. After bloodletting, or large loss of blood, the last that flows is of a brighter hue. In different periods of disease this florid blood is seen, and it has been attributed to the arterial blood passing rapidly through the parenchyma, without undergoing any change, or divesting itself of its principles for the purposes of nutrition, which latter function is found to be considerably impaired. Stevens attributes the changes in the blood to the different proportions in which the neutral salts which it contains, may exist; Rossi, to the influence of electricity.

*Influence of the Section of the Nerves on the coloration of the Blood.—Several theories.*

The venous blood may assume a darker hue, and become as black as ink. The venous blood has undergone this change by the introduction of a poison, such as the miasmata of typhus, &c. In some epidemics, according to Stevens, the blood presented that dark or black colour in persons in whom the epidemic disease had not as yet shown any of its symptoms; and according to the same author, this blood, on exposure to the air, did not become red. In the cholera the blood was black; in scorbutic patients, the blood is found of a darker hue. The blood may become yellow, and more especially its serum, without its containing any bile; animals stung by scorpions exhibit this appearance. If the blood becomes green by the addition of nitric acid, bile is present. The blood has sometimes presented a white or milky colour, or merely contained streaks of white, which were supposed to be derived from the presence of chyle or milk; but this whitish colour is produced by a greasy matter, described by Chevreul.

The buffy coat differs in colour from the crassamentum. It varies in thickness from one to nine lines, and is modified by the vessel which receives it. When thin, it may be florid or greenish; when thick, white or yellowish. Its consistence does not depend upon its thickness. When soft on its surface, it frequently contains mucus; when well formed, the crassamentum on which it rests is very soft; may be partial or general, or cellular; as large as the crassamentum, or greater or less in size. Its shape may be flat, like the crassamentum, or it may be hollowed in the centre, and raised at the edges (cupped); may be perfect or imperfect. The appearance of the buffy coat, when perfect, is of importance; but when imperfect, much value should not be attached to it, as it is met with in many dissimilar diseases. The buffy coat is composed of the fibrine, sometimes containing in its meshes serum and albumen; and on its surface is sometimes found a softish substance, which appears to be altered albumen.

The buffy coat, in its formation, is influenced by certain physical causes. Blood drawn from a vein will present a buffy coat, while the same, drawn, by cupping, from the capillary vessels, will not present a trace of it. According to the different forms of the vessels into which it is received, it will be present or wanting; that drawn from a small orifice will not present it, and, if allowed to trickle down the arm, the same results will happen; though this rule does not hold good in all cases. If the bleeding be interrupted by a state of syncope, that which flows after the syncope will not be buffed. If the blood be well shaken it will prevent its buffing; and the same will happen if it be allowed to fall down from a height. If received into vessels which are large and flat, the buffy coat is with difficulty formed; whereas, conical and narrow vessels favour greatly its formation.

*Influence of diseases on the formation of the buffy coat.*

If the character or disposition of the disease hinder or is unfavourable to its formation, we cannot promote it by any physical influences; and in those cases where there is a disposition to its formation, it will make its appearance although opposed by the same influences.

The importance of the buffy coat has been admitted by some, and denied by others. Both these sects are in error. The buffy coat does not always imply the existence of an inflammatory state.

I have preserved notes of 1494 cases in which bleeding had been practised, the blood having been drawn from orifices of the ordinary size, and been received into vessels of the same size and shape. The following are the results:—

| | |
|---|---|
| Perfect buffy coat, | in 643 of these cases. |
| Imperfect do. | „ 183 „ „ |
| No appearance of do. | „ 668 „ „ |

The buffy coat may be absent in all the different varieties of diseases. In its perfect form it is most frequently met with in the following cases:—

| | |
|---|---|
| Acute articular rheum. | in 4-5ths of cases. |
| „ pneumonia, | „ „ „ |
| „ pleurisy, | „ „ „ |
| Pulmonary tubercles, | „ „ „ |
| Acute metro-peritonitis, | „ „ „ |

In the first stage of pulmonary tuberculisation, the blood does not present this coat, but, as the stages of this disease are more advanced, it becomes more frequent; and when caverns have been formed, the buffy coat is as often met with as in pneumonia, but its thickness is less. Next in order to these diseases comes erysipelas of the face, in which the buffy coat is found perfect in three-fifths of the cases; imperfect in one-fifth; and absent in one-fifth. In angina and amygdalitis, it is found perfect in one-half of the cases only. Taking peritonitis and nephritis together, it is as constant in them as in pneumonia. In painter's colic, it occurs in one-third of the cases; and in pulmonary emphysema and ophthalmia it is found in the same proportions.

Bronchitis, even when capillary, does not exhibit it in more than one-fourth of the cases, and the same proportion answers for gastro-enteritis and chronic rheumatism. In effusions into the pleura after fever, in intermittent fevers, organic affections of the heart, and cerebral congestions, it occurs in the proportion of one-sixth of the cases. In cerebral and uterine affections, in one-seventh, and in lumbago, in one-tenth of the cases. In the latter affection, the imperfect one is more frequent than the perfect one, and shows itself in one-third of the cases. In the cholera, the buffy coat was absent when advanced to the period of cyanosis. In hysteria, sciatica, pleurodynia, simple jaundice, it was either imperfect or absent; and not met with in neurosis, mental alienations, chorea, delirium tremens, saturnine epilepsy, facial neuralgia, simple or mercurial erythismus, scurvy, &c.

I have not many cases of scarlatina, measles, or small-pox, in which I have employed bleeding. In scarlatina I have never seen the blood with a perfect buffy coat, but frequently with an imperfect one; in measles, very seldom; and in small-pox I have found the buffy coat as the general rule, and I would place it in frequency after pneumonia, but my data are not sufficiently numerous, and the appearances differ according to the stages of the disease.

In speaking of small-pox we must consider three circumstances: first, whether there be a complication of pneumonia or of pus in the blood; second, whether the eruption be distinct or confluent, (all those cases in which the buffy coat was well developed, were of a confluent character;) third, period of the disease: before the appearance of the eruption, I found, as in typhus fever, no buffy coat; but I must say that I have bled but in a very small number of cases. After the appearance of the eruption, from the second to the fith day, I have found a firm and thick buffy coat, similar to that of acute rheumatism. At the period when the pustules become filled with pus, and when the fever of suppuration is established, the buffy coat still showed itself, but becoming soft, thin, and imperfect, and covered with a kind of infiltrated mucus.

The buffy coat is imperfect or absent in epistaxis, blenorrhagia, systitis, erythema, urticaria, and venereal buboes. I have never found it perfect in any of these diseases.

In chlorosis of a pure, simple, uncomplicated character, I have bled three times. In the first case I found the blood presenting a perfect buffy coat, thick, firm, consistent, with elevated edges, and, in this case, there was no fever—the crassamentum was small, with a large proportion of serum. In the second case the blood was imperfectly buffed, and, in the third case, was not so at all. In another case of chlorosis, complicated with acute articular rheumatism, the blood was covered with a perfect coat.

It has been asserted that the blood drawn from a horse is always buffed and cupped; but that drawn from a healthy man is not so. De Haen and Boreski did not attach any importance to this coat, and denied its value. Authors often confound the part of the blood which becomes oxygenated with the buffy coat. Thomasini attaches great importance to this appearance of the blood being buffed; and says it is to be met with in a circumscribed phlegmasia, or one of a general character, or in a phlogistic diathesis, such as is presented by a pregnant woman. He considers chlorosis as a phlogosis of the blood-vessels.

The blood may be buffed in all diseases; its value depends upon the proportion in which it is found in such diseases, and depends more upon a certain condition of the blood than upon any particular disease. This condition of the blood may be met with in different diseases, and may thus explain its appearances in such and such cases. I merely give this explanation, without attaching much value to it.

*Influence of the febrile state upon the presence or absence of the buffy coat.*

Fever alone will not produce it—witness typhus; however, we generally find fever accompanying its presence; for it does not exist in acute articular rheumatism unless fever be present. Plethora alone will not produce it. The constitution has no influence over its production. Does the complex phenomenon called inflammation, possess any influence over its formation? Those diseases which present the best-marked symptoms of inflammation are also those in which it is found most perfect. Its existence alone will not reveal the nature of the disease; and its presence does not furnish a reason for bleeding; for in phthisis it is always to be met with. In acute articular rheumatism, after twenty blood-lettings, it will still be found as long as the intensity of the disease lasts, and although the crassamentum of the blood becomes less.

The mechanism of the formation of the buffy coat is wrapped in the same mystery as the cause which produces it. Its formation is prevented, or with difficulty effected when the blood coagulates too fast. If the blood does not coagulate, it will not be produced, and it is not necessary for its formation that the same condition of the blood should always exist. It may be formed under different conditions of the blood.

*Theory of its formation.*—The quantity of fibrine in the blood is increased, and, if the coagulation takes place slowly, the fibrine becomes completely separated, and, its specific gravity being less than that of the globules, it floats on the top, forming there the buffy coat. M. Denis describes fibrine as a modification of albumen. The buffy coat is composed of a portion of the fibrine which separates from the globules. Why and by what means produced? We can give no answer.

*Alterations of the Odour of the Blood.*

Morton describes a patient whose blood was stinking. Morgagni relates cases where it presented an acid odour. Stevens detects the yellow fever by the odour of the blood. I have never been able to discover any smell in the blood of small-pox or typhus patients. Poisonous substanees might communicate their odour to the blood as they are carried along with it.

*Alterations of the Taste of Blood.*

In the cholera the blood becomes insipid. The ancients said that, in Rachitis, it was acid.

*Heat of the Blood.*

In some diseases the temperature becomes elevated in several degrees, and it remains still to be seen whether the blood partakes in this elevation of temperature. It was asserted that, in the cholera, its heat fell three or four degrees, and in the cold stages of intermittent fever, this lowering of the temperature had been noticed. I deny this fact; for I have seen, in the cold stage, the thermometer indicating a temperature equal to and even above the natural standard of a healthy man. In hysteria, where the nervous system is so much disturbed, we might expect to find the heat of the blood decreased. Morgagni relates a case of a young hysterical woman, from whom the blood drawn was cold. I am not aware of any authenticated case in medicine, in which the temperature of the blood was increased.

*Electricity of the Blood.*

According to Bellingheri, in what he calls inflammatory diseases, the blood drawn from a patient is deficient in electricity; and, according as the symptoms of inflammation are on the increase, the electricity of the blood decreases, and *vice versâ.* The electricity would be negative when the blood was flowing and was buffed, and would be less than in the healthy fluid. When the electricity was in great quantity, there would be no buffy coat; its electricity would be greater at the beginning than at the close of a bleeding; but on all those subjects science is still in its infancy.

*Alterations in its Consistence.*

Its consistence may increase to the extent either of becoming thicker or of becoming solid. It may be diminished, becoming very liquid, not forming any clot, when drawn from the body, or when found in the dead subject.

*Augmentation of its Consistence.*

The doctrine of Boerhaave was based upon the thickness of the blood, which, according to him, was such as to prevent its molecules from passing through the minute capillary vessels; thus producing a congestion, depending either upon this thickened condition or upon some change in other respects. In fevers, the blood becomes thickened by the escape of the liquid or watery parts, and by the increased temperature. Hoffman considered these obstructions produced by the thickened state of the blood, as giving rise to fever, and the conversion of the blood into pus. All those opinions are merely theoretic, being unsupported by proofs, and were upset by the solidists. At present those ideas are again brought forward; and Magendie has experimented on this subject. Substances which, mixed with blood, thicken it—such as oil, grease, &c.—were introduced into the veins of animals who died with either symptoms of pulmonary affections, or death was produced slowly by other causes. On examination, he found congestions and extravasations of blood—engorgements resembling pneumonia in its first or second stages. Such occurrences may be explained by supposing these foreign substances incapable of passing through the lungs, and thus producing congestions. If charcoal be injected into the veins of an animal, he recovers from its effects, as the molecules of the charcoal, reduced to an impalpable power, are smaller and mingle with the blood without thickening it. The blood may thicken spontaneously, and, by analogy, we may believe that it may become so to such degree as will prevent its passage through the vessels, and congestions thus be formed: experience, however, does not prove this. In one case, by the aid of the microscope, was seen a portion of blood thickened, endeavouring to pass through, and stopped before the capillary vessels, until propelled through them by a violent contraction of the heart produced by the agitation of the animal. I am of opinion that a great number of inflammatory congestions may be accounted for by the thickened condition of the blood rendered thus incapable of passing through the smaller vessels: this opinion may, perhaps, one day be borne out by future researches.

Boerhaave thought that some kinds of food thickened the blood; and he is supported in this theory by some facts in physiology—as such a food producing a milky chyle, and another kind furnishing one of a different character. May these different chyles produce differences in the blood? We know not. Many diseases seem to be cured by diluting the blood by means of drinks. Some skin diseases are thus removed. Water injected into the current of the blood separates itself, passing off either by absorption or through the kidneys. Water given to a sick person passes off by excretion, it being very difficult to dilute the blood, as this fluid, by a law of its own, endeavours to repel it: the blood is naturally viscous; its viscosity increases with the augmentation of its consistency. In the cholera its viscosity was increased, and the water of the serum diminished. The greater viscosity of the blood in inflammation has been attributed to the large quantity of albumen which it contains. The serum may become viscous, or its viscosity may diminish.

*Coagulation of the blood during life time.*

We find clots in the heart, if we open the body fifteen hours after death, before putrefaction has commenced. In animals, we find them one hour after death; if we delay till twenty-four hours have elapsed, our chances of finding them are lessened, as the blood liquifies when putrefaction sets in. There are some cases in which the clots found after death have been formed during life time—can we detect their formation in the living subject? The clots differ much from each other, and are found red, white, red externally, white internally, or white externally and red internally, or striated with red and white; these streaks have been taken for vessels, which is erroneous. Their consistence may be firm or soft, dry or infiltrated, and easily torn; their size may be large, occupying all the heart, and branching into the arteries and veins. The clots formed after death may contract from adhesions with the walls of the heart internally. In the hearts of animals examined just after death, no clots are found; but in a few hours after, we find them formed and adhering to the walls; around the clot is found a pellicle resembling a membrane, and which may easily be mistaken for a false one, although it is merely a physical phenomenon, resembling the perfect buffy coat. Some clots are formed in the heart during life time—are their characters similar to those formed after death? We can distinguish them by certain signs—but are those signs well marked? They are not; the physical peculiarities are not sure enough. If the clots do not present characters which serve to distinguish them from those which are found in almost all the hearts which we examine after death, I cannot place reliance upon the diagnosis which pretends to ascertain their presence during life. How can the blood, so agitated as it is in the heart, coagulate during life time?

The friability of these clots, and their gray colour, have been given as proofs of their being found during life: at the summit of the valves, when they are disorganized and rough, we find polypi which appear composed of several layers, and presenting a degree of organization. The best signs of their formation during life, are their being organized in their centres; but this we find very rarely.

I have injected the clots found in the heart, by the coronary arteries, and their being thus injected was considered as proving the existence of vessels in them; but I do not look upon it as such a certain sign. The existence of pus in their centre, was advanced as another such proof; but has it been secreted there? Before we can admit it, we must allow the organization, the inflammation, and the power of secretion of the clot, which is far from being proved; for it may be formed by the endocarde in a state of disease, or carried into the heart with the blood, and at death has become separated from it, and afterwards surrounded or inclosed by it during its coagulation. I have never found pus in a clot which I could assure myself had been secreted there: clots may form during life in those cases which present some alteration of the lining membrane, a molecule of blood attaching itself to some part of its altered surface, and there showing a tendency to conglomerate; this disposition may be increased by a condition of too great viscosity of the blood, and they have been found when the smoothness of the lining membrane has been altered.

The alteration which we now describe under the name of dissolution of the blood, formerly was called putridity. It may exist in different degrees: in the one, the clot, although distinct from the serum, is so soft as to be readily broken down, and mixes freely with the serum under a slight touch of the finger; in the other, the clot is absent, and a reddish liquid is found, with grumous masses floating about in the reddened serum. We must not confound the dissolution of the blood where the solid parts of that fluid remain uncoagulated, with that condition in which the coagulable principles of the blood are absent entirely or in part, producing a clot of small dimensions floating in a large proportion of serum: in the state of dissolution, the solid parts of the blood merely form grumous masses mixed and confounded with the serum.

This condition of the blood may exist in different classes of cases: in one variety, the cause which produces it is evident, as when a concentrated solution of sub-carbonate of soda was injected into the veins of an animal, by Magendie; the blood was changed into a liquid of a reddish colour, containing portions of fibrine separated into grumous parcels. Does the blood become more alkaline where this state of dissolution exists? the following facts have been given as proofs. In blood of a scorbutic patient, which was not coagulated, there existed a larger proportion of soda than natural. In a very severe case of typhus fever, Denys detected the presence of free ammonia and of an ammoniacal salt.

Bretonneau describes mercury as producing a dissolution, but this is not proved, and the same effect is caused, according to Grange, by the exhibition of resinous purgatives, cicuta, and laurel water. The sulphuric, nitric, and prussic acids, mixed with the blood in vessels, dissolve it; also some gases, as the sulphuretted hydrogen.

*Influence of pus.*—It has been said to coagulate the blood which is found in an inflamed vein; it varies in its properties, being either acid or alkaline, sour or sweet, forming a bloody liquid or a thick one—and according to these different conditions it may produce corresponding effects: blood is not changed by the mixture of a laudable pus, but if mixed with serous pus, it is dissolved, and is prevented from coagulating. In some cases of resorption of pus, I have found the blood in a state of dissolution in the heart and blood-vessels; in other cases I have found clots in the heart, &c. Gaspard has dissolved the blood by injecting putrid matter into the veins: can this condition be produced by the introduction into the blood of imperceptible miasmata, and is it owing to their pressure that the blood in the plague and cholera is found dissolved? Do the pernicious intermittent fevers depend upon this altered condition of this fluid? The state of the blood in these latter diseases has not been ascertained.

Millman says, that in scurvy the blood is not changed; in the great majority of cases it is found dissolved. According to L'Inde, the blood is not changed in the first stage of this disease, but as it advances in its progress it becomes more and more altered until it becomes entirely changed into a reddish fluid. I am of opinion that miasmata are injurious by producing certain changes in the blood, which are not discoverable in the first days of the diseases.

*Viruses.*—In small-pox, the blood has sometimes presented an imperfect buffy coat, and in the severe forms of it, it has been found dissolved. Is this owing to the presence of pus? The hydrophobic virus has been mentioned as producing the dissolution of it: I cannot give any opinion on this subject. Can it be produced by the food employed? I think, that an insufficiency of aliment, combined with bad air, may produce the scurvy: a humid atmosphere has been mentioned as a cause of the dissolution of the blood, which state, however, must be distinguished from its mere liquidity. The blood may exert influence over the nervous system, which may be differently excited by it, according to its various morbid conditions: this is a very serious qustion, and one very difficult to decide.

Dupuy, of the veterinary school of Alfort, has made experiments on this subject. After the section of the eighth pair of nerves, the blood has been uncoagulated: the function of hæmatose being by this section disordered, may it not account for this result? Meyer, on the other hand, did not find its coagulability impeded by this section. Authors assert that a violent commotion of the spinal marrow has produced this condition of dissolution: persons struck by lightning have presented the same appearance of dissolution. An animal tired to death has his blood in a dissolved state; purpura hæmorrhagica, a disease in which the blood has been found dissolved, may develope itself independent of any of those causes which we have as yet examined. I believe that in former times the dissolution of the blood, owing to various causes, was more common than at present, as may be seen by the description of the blood by the old authors, and the diseases which then prevailed.

*Consideration of the causes which tended to produce this condition of the blood.*

In the middle ages they were much more numerous, the habitations of men being more crowded together, the circulation of pure air more impeded, and the individuals more closely packed together; at the same that the food was of a less wholesome quality, famine more frequent, the towns badly built, with narrow streets never visited by the sun, the supply of water deficient, noxious and impure in quality, &c. These sources of disease having been removed, we meet much more rarely in the present day, those diseases which produce the dissolution of the blood.

The writings of the 15th, 16th, and 17th centuries contain numbers of cases in which the blood was dissolved, or coagulated very imperfectly, and many diseases characterized by this condition of the blood, such as scurvy, spontaneous gangrene, and purpura, are much rarer at present. The typhus fever which raged in Ireland some years ago, presented many of the features of those diseases which are described as belonging to the middle ages, and which are rarely met with at present, owing to the improved condition of the people: their existence at the present day among the people of Ireland may be attributed to the events and poverty with which that unhappy people is afflicted, and which are not be paralleled in any other part of Europe.

The state of the dissolution of the blood produces symptoms resembling those of poisoning, and is very favourable to hæmorrhages: when the blood is merely in a liquid condition, dropsies but not hæmorrhages are produced: the secretions may be altered when the blood is dissolved, and tumefaction of the spleen is a very constant attendant in these cases. I do not think that the blood during life can pass into a state of putridity, such as has been described by old authors.

We have so far described all those alterations of the blood which are recognisable by our senses, and we shall next describe those which are revealed to us by the microscope.—*London Medical Gazette.*

---

*On Penetrating Wounds of the Heart.* By J. A. Jobert.—Three interesting cases of penetrating wounds of the heart are related, where the persons lived after the receipt of the injury from twelve hours to no less than ten days. In all the cases the wound of the heart was indicated by a peculiar whizzing sound, precisely similar to that which exists in varicose aneurism; and from its presence being recognised in all these three cases, M. Jobert thinks it might be regarded as a diagnostic mark of the presence of a penetrating wound of the heart. He relates several cases which have been published by other authors, where patients have lived for years after this organ had been wounded, opportunities having in many of these cases occurred, of confirming the diagnosis by examination after death. He regards, therefore, a wound of the heart as not necessarily fatal, and gives numerous directions of the mode in which the treatment may be best conducted. Of these, bleeding, both general and local, the local application of cold, and the administration of such remedies as tend to reduce the force of the circulation, and abate the tendency to hæmorrhage, are the chief.—*Archives Generales*, Sept. 1839.

---

*Periodical Uterine Dropsy.*—The patient was a married woman, 32 years of age, of lymphatic temperament, and her constitution impaired by hard work, bad food, and unhealthy residence. Every month she passed *per vaginum* from twenty-five to thirty pints of clear, limpid water, of a slight yellowish tinge, and for two days after a few drops of blood, deficient in colouring matter. After this evacuation she was perfectly well; but another collection of water soon formed, of which she was sure to be relieved the following month. This condition had continued eight months before she was seen by Dr. Dubedat. She could assign no cause for the disease. It had come on without injury or sensible lesion of any kind, or alteration of their functions. The only change in her health was emaciation and general debility, which was daily increased. The abdomen was of extraordinary size, and there was considerable dyspnœa; the pulsations of the heart were regular; the digestion was bad; and the bowels were constipated. The urine was scanty, but paosed frequently, yet without pain; the pulse was feeble, slow, and contracted; the face was pale, as were also the tongue and gums; and there was considerable blueness around the eyes.

On examination, the body of the uterus was found to be much enlarged and infiltrated, yet light compared with its size; its neck was also infiltrated and shortened.

To relieve the constipation, castor oil was administered, followed by emollient enemata, and, as drink, barley-water with nitre.

The next day pains in the loins were felt, announcing that an evacuation of water was about to take place; and during that night water was discharged so abundantly as to inundate the bed.

Three days after the abdominal swelling was gone, the digestion had improved; her appetite returned; the pulse was more natural, and her menses had ceased.

She was put on a course of diuretics; nevertheless, in a fortnight the abdomen had enlarged, and continued to increase till again evacuated in the same manner, followed by the menstrual discharge; the quantity of water evacuated was, however, only about half what had come on the former occasion. The diuretic medicines were then given in increased doses, and afterwards followed by tonics, and under this treatment the watery discharges became less frequent and were diminished in quantity; the menstrual discharge, too, returned of a more natural appearance, and in a few months she was free from complaint.—*Bulletin General de Therapeutique*, May, 1838.

---

*Mortality from Phthisis, at Naples.*—In a late number of the Filiatre Sebizio, M. de Renzi controverts the conclusions concerning the mortality by phthisis, at Naples, to be found in a paper presented by M. Journé to the Academy of Medicine at Paris. The sum total of phthisical patients received into the Hospital of Incurables during the years 1835-36 and '37, is reduced by M. de Renzi from 2969 to 2775; and he shows that of this number only 1846 belonged to Naples and its suburbs, the rest consisting of strangers who come to die there. This gives an average of 615 instead of 989, which was M. Journé's supposition—a considerable difference, particularly if the probabilities of medicine are to be based on figures. Then subtracting the catarrhal affections, or those which resemble phthisis, M. de Renzi fixes the number of phthisical patients at 600, which, in a population of 400,000, gives one in 666. We must add, however, 200 patients belonging to the middle and upper classes, which will make 800; and of these 560 die, or 70 per cent. But, as the ordinary mortality is 13,000, the proportion of deaths from phthisis is about 1 in 23.

The mean mortality at the Hospital of Incurables is 1800, and the number of deaths among the phthisical patients belonging to the town is 430; so that these deaths form a quarter of the total mortality in the institution. But if we consider, *first*, that at Paris more than a third of all patients are treated in the hospitals, and hardly a fifth at Naples; *secondly*, that at Paris, the number of consumptive patients received is equal to those suffering under other diseases, while at Naples the latter are much fewer in proportion, it will be allowed that a comparison is difficult. This being settled, if in the hospitals of Paris, as Bayle has demonstrated, and M. Journé laid down, one-third of the patients die of phthisis, and this number is more than a third of all the deaths in the whole town; if, on the other hand, in the hospitals at Naples, less than a quarter of the patients die by phthisis, and if, even adding the patients who do not belong to the town, we do not reach a third, as at Paris; if the phthisical patients in the hospital include nearly all in town, whilst the deaths in the hospital are but a sixth of the general mortality, it necessarily follows that the patients who die of phthisis are in proportion to those who die of other diseases, as 1 to 4 at Paris, and 1 to 12 at Naples. Hence the climate of Naples, far from favouring the development of the disease, makes it rarer than it would naturally be in a town of small size, relatively to its population, who are in consequence crowded. If we consider, moreover, that scrofulous affections are very common there, we must necessarily conclude, that the number of consumptive patients does not exceed the natural proportion to those afflicted with scrofula.

As to the important question, whether a phthisical patient from a cold or northern climate may experience benefit in a more temperate one, M. de Renzi answers it in the affirmative, and says he knows it from his own experience. But if the disease has already made great progress, if the disorganization of the lungs is much advanced, and the tubercles have begun to soften, a southern climate is of no avail. In such cases the poor patient travels but to perish on a foreign shore, far from his reiatives and friends.

*Gazette Medicale*, Decem. 29, 1839.

